# *Lats1/2*-Mediated Alteration of Hippo Signaling Pathway Regulates the Fate of Bone Marrow-Derived Mesenchymal Stem Cells

**DOI:** 10.1155/2018/4387932

**Published:** 2018-12-24

**Authors:** Lang Li, Liang Dong, Yifeng Wang, Xiuhong Zhang, Jie Yan

**Affiliations:** ^1^Intensive Care Unit, The Affiliated Wuxi People's Hospital with Nanjing Medical University, Wuxi 214023, Jiangsu, China; ^2^Department of Pharmacy, The Affiliated Wuxi People's Hospital with Nanjing Medical University, Wuxi 214023, Jiangsu, China

## Abstract

Bone marrow-derived mesenchymal stem cells (BMSCs) can be used to enhance lung repair in acute respiratory distress syndrome (ARDS); however, the repairing effect is limited by poor homing and retention of BMSCs. The purpose of this study was to investigate whether* Lats1* and* Lats2*-mediated alteration of Hippo signaling pathway could promote the differentiation, proliferation, and migration of BMSCs. BMSCs were transduced by lentiviral vectors for high and low expression of* Lats1* and* Lats2*. The expression levels of* Lats1*,* Lats2*, YAP, and 14-3-3, respectively, were assessed to clarify the regulatory effects of* Lats1* and* Lats2* on Hippo signaling. Osteogenic (Runx2 and OSX) and adipogenic (C/EBP*α* and PPAR-*γ*) transcription factors were determined to clarify the effects of Hippo signaling on BMSCs differentiation. The effects of Hippo signaling on BMSCs proliferation and horizontal and vertical migration were also measured by CCK-8, scratch assay, and Transwell migration assay, respectively. Lentiviral transduction efficiency could reach 93.11%–97.14%. High and low expression of* Lats1* and* Lats2* could activate and inhibit the Hippo signaling pathway, respectively. High and low expression of* Lats1* and* Lats2* could inhibit and promote BMSCs differentiation into osteoblasts and adipocytes. High and low expression of* Lats1* and* Lats2* could inhibit and promote BMSCs proliferation and horizontal and vertical migration, respectively. Our studies suggest that* Lats1*/*2*-meidiated inhibition of Hippo signaling in BMSCs may optimize their effects of tissue repair in ARDS, suggesting a novel strategy for enhancing disease therapeutics.

## 1. Introduction

Acute respiratory distress syndrome (ARDS) constitutes the acute noncardiogenic refractory respiratory failure resulting from injured alveolar epithelial cells [1]. Therefore, stem cells-based repair of damaged alveolar epithelial cells is highly desirable for the treatment of ARDS [2]. Recent studies have shown that mesenchymal stem cells (MSCs) can be used as seed cells for lung repair in ARDS, as they can differentiate into alveolar epithelium, alleviating lung inflammation and improving survival in animal models of ARDS [3, 4]. However, difficulties such as low percentages of MSCs homing to ARDS lung tissues and short MSCs retention remain to be addressed. Therefore, it is important to clarify the regulatory mechanisms of MSCs differentiation, proliferation, and migration [5, 6]. Notably, the Hippo signaling pathway is able to regulate the differentiation, proliferation, and migration of many cell types; however, its role in MSC regulation remains to be elucidated [7, 8].

In this study, we utilised lentiviral vectors to modulate the expression of Hippo signaling regulators, large tumour suppressor 1 (*Lats1*), and large tumour suppressor 2 (*Lats2*), in bone marrow-derived MSCs (BMSCs). We also investigated the effects and possible mechanism of* Lats1*/*2*-mediated alteration of Hippo signaling pathway on the differentiation, proliferation, and migration of BMSCs.

## 2. Materials and Methods

### 2.1. Main Materials and Reagents

Gateway BP Clonase II Enzyme mix and Gateway LR Clonase II Enzyme mix were purchased from Invitrogen (Carlsbad, CA, USA); Mini Plasmid Kit was purchased from Tiangen Biotech (Beijing, China); 10% foetal bovine serum (FBS), Dulbecco's modified Eagle's medium, (DMEM)/F12 medium, DMEM medium, the full length coding sequence of* Lats1 *(NM_010690.1, 3390 bp) and* Lats2 *(NM_015771.2, 5213 bp), and the 293FT cell line were purchased from Thermo Fisher (Waltham, MA, USA); C57BL/6 mouse BMSCs, C57BL/6 BMSCs osteoblast differentiation induction kit, C57BL/6 BMSCs adipocyte differentiation induction kit, and the lentiviral expression vector pLV.EX3d.P/neo were purchased from Cyagen Biosciences Inc. (Guangzhou, China); mouse Anti-mouse GFP antibody and Cell Counting Kit-8 (CCK-8) were purchased from Abcam (Cambridge, MA, USA); BCA protein quantitation kit, RIPA cell lysis buffer, nuclear and cytoplasmic protein extraction kit, and horseradish peroxidase- (HRP-) conjugated goat anti-mouse IgG were purchased from Beyotime Biotechnology (Nanjing, China); rabbit anti-*Lats1* antibody, rabbit anti-*Lats2* antibody, rabbit anti-YAP antibody, rabbit anti-14-3-3 antibody, rabbit anti-*β*-actin antibody, and rabbit anti-GAPDH antibody were purchased from Santa Cruz Biotechnology (Dallas, TX, USA); HpaI, XhoI endonucleases, RNA extraction kit, real-time qRT-PCR detection kit, and PCR primer sequences (itemized below) were purchased from TaKaRa (Otsu, Japan).

### 2.2. Cell Culture and Identification

All protocols involving the use of animals have been approved by the Institutional Animal Care and Use Committee (IACUC) at Nanjing Medical University. Commercially available C57BL/6 mouse BMSCs and the 293FT cell line were purchased as described and used in this study. The phenotypes of BMSCs were verified by the supplier using flow cytometry and their identity was determined based on their ability to differentiate into adipocytes, osteoblasts, and chondrocyte. BMSCs and 293FT cells were cultured in DMEM/F12 supplemented with 10% FBS, 1% streptomycin, and 1% penicillin in a 37°C, 5% CO_2_ incubator.

### 2.3. Recombinant Lentiviral Vector Construction and Packaging

The Gateway recombination cloning technique was adopted along with the lentiviral vector containing double EF1*α* promoters for overexpression to construct recombinant lentiviral vectors highly expressing* Lats1* and* Lats2*, respectively. The full-length coding sequences of* Lats1* and* Lats2* were cloned into the lentiviral expression vectors pLV.EX3d.P/neo via the BP and LR reactions and were positioned between the EF1*α* promoter and internal ribosome entry site- (IRES-) dependent enhanced GFP (eGFP), to successfully construct lentiviral vectors EF1*α*-*Lats1*-IRES-eGFP highly expressing* Lats1* and* Lats2*, respectively. The vector EF1*α*-eGFP was used as an empty vector control. Based on the* Lats1* gene sequence (NM_010690.1) and* Lats2* gene sequence (NM _015771.2) in GenBank and the principles of RNA interference, the online software Block-IT RNAi Designer (Invitrogen) was used to design shRNA fused with eGFP specific for* Lats1 and Lats2*, as well as a positive control sequence to be carried in the lentiviral vector pLLU2G. Primers were synthesized by Invitrogen, and the single-stranded primers were annealed into a double-stranded oligo sequence, which was then ligated into the linearized RNAi expression vector that had been digested with both HpaI and XhoI endonucleases. The* Lats1* shRNA sequence was 5′-GCACACATCATAAAGCCTTGC-3′ and the* Lats2* shRNA sequence was 5′-TCAACGTGGACCTGTATGA-3′. After sequence verification, plasmids pLP1, pLP2, and pLP/VSVG were cotransfected into 293FT cells using the viral packaging and transfection reagents for lentiviral packaging. After culturing for 48 and 72 h, the culture medium was collected. Viral particles were collected using a centrifugal ultrafiltration device and were concentrated, being subsequently diluted with DMEM supplemented with 10% FBS to transfect 293FT cells. The viral titre was determined using serial dilution and the levels of GFP expression.

### 2.4. Lentiviral Vector Transduction of BMSCs and Detection of the eGFP Reporter

BMSCs were seeded into a 6-well plate at the density of 2 × 10^5^/ well. Transduction was carried out by adding 1.0 ml DMEM/F12 supplemented with 10% FBS and lentiviral vectors to the cells, which were mixed well and placed in a 37°C, 5% CO_2_ incubator overnight. After 12 h, culture medium containing lentivirus was replaced with normal culture medium. Experimental design included the normal control group (MSC group), the empty vector control group (MSC-GFP), the* Lats1* overexpression group (MSC-*Lats1*), the* Lats2* overexpression group (MSC-*Lats2*), the empty vector for RNAi group (MSC-shcontrol), the* Lats1* RNAi group (MSC-sh*Lats1*), and the* Lats2* RNAi group (MSC-sh*Lats2*). The transduction efficiency was determined using a BX43 fluorescence microscope (Olympus, Tokyo, Japan) and the percentage of eGFP-positive cells was quantified by FACSCanto II flow cytometry system (BD Biosciences, San Jose, CA, USA).

### 2.5. Western Blot Detection of* Lats1*,* Lats2*, and YAP Protein Expression Levels

Proteins were extracted using the cytoplasmic protein extraction kit. Protein concentrations were determined using the BCA method. After protein denaturation, 25 *µ*l protein sample was loaded into each well for sodium dodecyl sulphate-polyacrylamide gel electrophoresis analysis, which was then transferred to the polyvinylidene fluoride membrane. Blocking was carried out using 5% skim milk in Tris-buffered saline with Tween 20 (TBST) for 1 h. Primary antibodies were added to the membrane, followed by incubation at 4°C overnight, and GAPDH or *β*-actin was used as an internal reference protein. Membranes were then washed with TBST three times. The HRP-conjugated secondary antibodies were added, followed by incubation at 25°C for 1 h. Membranes were then washed using TBST four times. Signals were detected using the enhanced chemiluminescence reagent and X-ray film exposure, and the films were scanned and the intensity (grayscale image) of each band was quantified using Quantity One software (Bio-Rad Laboratories, Berkeley, CA, USA).

### 2.6. Induction and Identification of Adipogenic and Osteogenic Differentiation of BMSCs

For osteoblastic differentiation, BMSCs were seeded into a 12-well plate, and 1.0 ml of DMEM/F12 supplemented with 10% FBS was added to each well. When the cell confluence reached 80–90%, the culture medium was replaced with osteoblast induction medium for continuous culture for 3 weeks. Calcium deposits were evaluated using Alizarin Red S staining followed by extraction with 10% cetylpyridinium chloride at 25°C for 15 min. The absorbance at 570 nm was then measured.

For adipogenic differentiation, BMSCs cells were seeded into a 24-well plate, and 1.0 ml of DMEM/F12 supplemented with 10% FBS was added to each well. When confluence reached 80–90%, the culture medium was replaced with adipocyte induction medium for continuous culture for 3 days. Then, the medium was replaced with the adipocyte maintenance medium for further culture for 24 h. After replacing the medium three times, cells were then cultured in the adipocyte maintenance medium for 1 week. Oil red O staining was used to evaluate the accumulation of neutral lipid vacuoles. Following isopropyl alcohol extraction at 25°C for 15 min, the absorbance at 520 nm was measured.

### 2.7. qRT-PCR Detection of* Lats1*,* Lats2*, YAP, Runx2, OSX, C/EBP*α*, and PPAR-*γ* mRNA Expression

Total RNA was extracted from BMSCs in culture plates using the TRIzol method. Total RNA (2 *μ*g) was added to the reverse transcription reaction to synthesize cDNA. An ABI Prism 7500 quantitative PCR instrument (Applied Biosystems, Foster City, CA, USA) was used for product amplification. The 2^−ΔΔCT^ method was used to calculate the relative expression levels of mRNA, and* GAPDH* was used as the internal reference gene. The PCR primers used were as follows:* Lats1*: forward primer, 5′CCA CCC TAC CCA AAA CAT CTG-3′, and reverse primer, 5′- CGC TGC TGA TGA GAT TTG AGT AC-3′;* Lats2:* forward primer, 5′- GCC AAA GAC TTT TCC TGC CA -3′, and reverse primer, 5′- TCT TTG CTC CCC AGG ACT TT -3′;* YAP*: forward primer, 5′-TGA ACA AAC GTC CAG CAA GAT AC-3′, and reverse primer, 5′-CAG CCC CCA AAA TGA ACA GTA G-3′;* Runx2*: forward primer, 5′-CCG TCA CCT CCA TCC TCT TTC-3′, and reverse primer, 5′-AAT ACG CAT CAC AAC AGC CAC A-3′;* OSX*: forward primer, 5′-ACC CTT CCC TCA CTC ATT TCC TG-3′, and reverse primer, 5′-TGC CTT GTA CCA CGA GCC ATA G-3′;* C/EBPα:* forward primer, 5′-CGG GAA CGC AAC AAC ATC GC-3′, and reverse primer, 5′-CGG TCA TTG TCA CTG GTC AAC TC-3′;* PPAR-γ*: forward primer, 5′- TCA GGC AGA TCG TCA CAG AGC-3′, reverse primer, 5′-TTG TCA GCG GGT GGG ACT TTC-3′; and* GAPDH*: forward primer, 5′-TAT GTC GTG GAG TCT ACT GGT-3′, and reverse primer, 5′- GAG TTG TCA TAT TTC TCG TGG-3′.

### 2.8. CCK-8 Assay for Determining Cell Proliferation

Cell proliferation was measured by the CCK-8 assay according to the manufacturer's instruction. BMSCs were resuspended in DMEM/F12 supplemented with 10% FBS to adjust the cell density to 2 × 10^3^/ml, and then cells (100 *µ*l/well) were incubated in 96-well plates. Cells were cultured for 1, 2, 3, 4, 5, 6, and 7 days before addition of 10 *µ*l CCK-8 solution to each well and the plates were incubated for 1 h at 37°C. The optical density of each well was measured at 450 nm using a MK3 microplate reader (Thermo Fisher, Waltham, MA, USA). There were six duplicate wells in each group and three independent experiments were performed.

### 2.9. Scratch Assay to Determine the Horizontal Migration of BMSCs

BMSCs were resuspended in DMEM/F12 containing 2% FBS, cell density was adjusted to 1 × 10^5^/ml, and 200 *μ*l BMSCs suspension was added to each well in a 6-well plate. When cells reached 80–90% confluence, a 10 *μ*l pipet tip was used to scratch the centre of the well, and the cells that were scratched off were washed off with PBS. DMEM/F12 without serum was added to each well, and the cells were cultured in a 37°C, 5% CO_2_ incubator for 24 h. Cells were photographed at 0 and 24 h of the incubation. The image files were opened using ImageJ software (National Library of Medicine, Bethesda, MD, USA), and five horizontal lines were randomly selected to calculate the mean width of the wound to reflect the degree of wound healing.

### 2.10. Transwell Migration Assay to Evaluate the Vertical Migration of BMSCs

BMSCs were resuspended in DMEM/F12, the cell density was adjusted to 1 × 10^4^/ml, and 200 *μ*l cell suspension was added to the Transwell insert (membrane diameter, 6.5 mm; pore size, 8 *μ*m) (Corning, Armonk, NY, U.S.A.) in a 24-well plate. Then, 600 *μ*l DMEM/F12 containing 10% FBS was added to the compartment beneath the Transwell insert. The Transwell migration assay was carried out in a 37°C, 5% CO_2_ in incubator for 10 h. Subsequently, cells on the upper layer of the Transwell membrane were removed using wet cotton swabs. Cells below the insert membrane were stained with 0.1% crystal violet solution for 20 min and were counted under an IX51 inverted phase-contrast microscope (Olympus. Tokyo, Japan). Five low-power fields were randomly selected to count the number of cells and the average cell number was calculated.

### 2.11. Statistical Analysis

Statistical analysis was performed using the SPSS 20.0 statistics software package (SPSS Inc., Chicago, IL, USA). Numeric data were presented as the means ± standard deviation ($\overset{-}{x}\pm s$). Comparisons of numeric data within groups or between groups were conducted using a t-test, analysis of variance (ANOVA), or a Mann-Whitney U Test. Categorical data were analyzed using a *χ*^2^ test. A* P* value < 0.05 was considered to be statistically significant.

## 3. Results

### 3.1. Identification and Efficiency of Lentiviral Transduction of BMSCs

**Figure 1:**
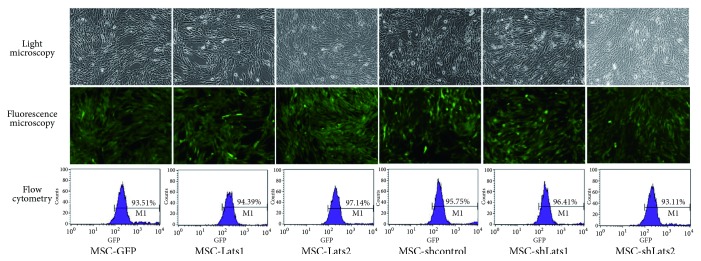
**Transduction efficiencies in BMSCs after lentiviral vector transduction**. The BMSCs were transduced with EF1*α*-eGFP (MSC-GFP), EF1*α*-*Lats1*-IRES-eGFP (MSC-*Lats1*), EF1*α*-*Lats2*IRES-eGFP (MSC-*Lats2*), pLLU2G–control (MSC-shcontrol), pLLU2G-sh*Lats1* (MSC-sh*Lats1*), and pLLU2G-sh*Lats2* (MSC-sh*Lats2*), respectively. The transduction efficiencies were determined by light microscopy (upper, 200×) and fluorescence microscopy (middle, 200×), and the percentages of eGFP-positive cells were measured by flow cytometry (bottom).

**Figure 2:**
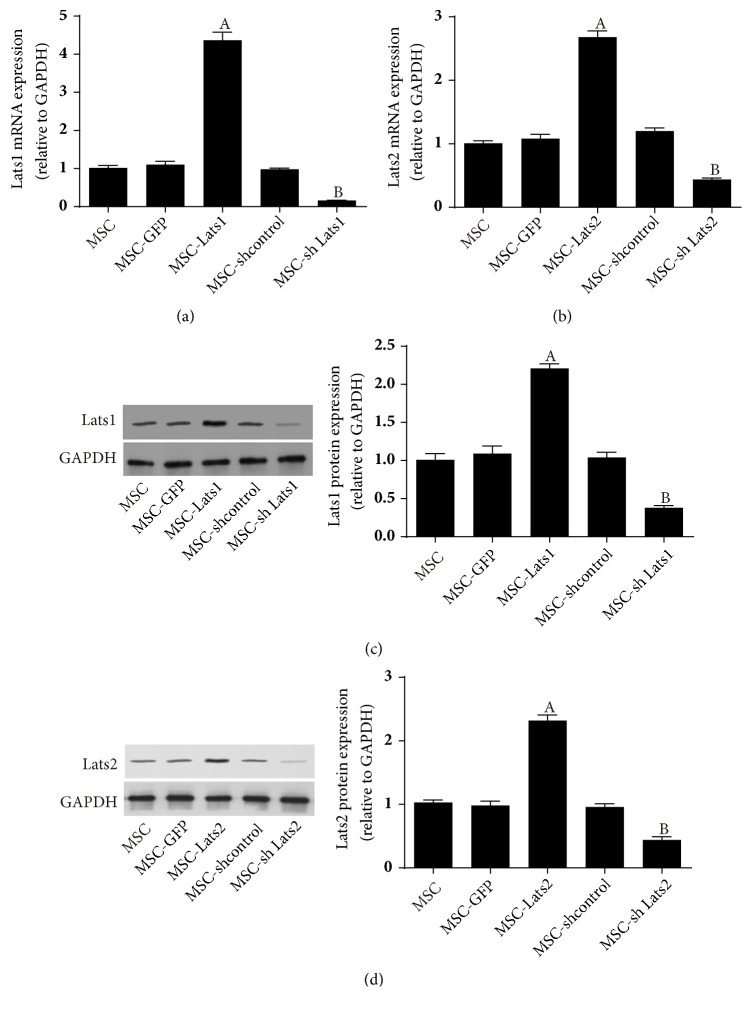
**Transgene expression levels of* Lats1* and* Lats2 *in BMSCs after lentiviral vector transduction**.* Lats1* (a) and* Lats2* (b) mRNA in BMSCs after transduction were evaluated by qRT-PCR. The expression of* Lats1* (c) and* Lats2* (d) protein in BMSCs after transduction were evaluated by Western blot (n = 3;  _ _^A^* P* < 0.05 vs. MSC-GFP,  _ _^B^* P* < 0.05 vs. MSC-shcontrol).

Fluorescence microscopy and flow cytometry were used to detect green fluorescent protein (GFP), and quantitative reverse transcription-polymerase chain reaction (qRT-PCR) and western blot were used to detect mRNA and protein levels of* Lats1 *and* Lats2*, respectively, to evaluate the efficiency of lentiviral transduction of BMSCs. The results indicated that the percentage of positively transduced cells by MSC-GFP, MSC-*Lats1*, MSC-*Lats2*, MSC-shcontrol, MSC-sh*Lats1, *and MSC-sh*Lats2* ranged between 93.11% and 97.14% (Figure 1). The* Lats1* mRNA expression level in the MSC-*Lats1* and MSC-group was significantly higher than that of the MSC-GFP group, whereas the* Lats1* mRNA expression level of the MSC-sh*Lats1* group was significantly lower than that of the MSC-shcontrol group (Figure 2(a)). The* Lats2* mRNA expression level in the MSC-*Lats2* group was significantly higher than that of the MSC-GFP group, whereas the* Lats2* mRNA expression level of the MSC-sh*Lats2* group was significantly lower than that of the MSC-shcontrol group (Figure 2(b)). However, there was no significant difference in the* Lats1* or* Lats2 *mRNA expression levels between the MSC, MSC-GFP, and MSC-shcontrol groups (Figures 2(a) and 2(b)). The analysis of* Lats1* and* Lats2* protein expression levels also yielded similar results (Figures 2(c) and 2(d)). The above results suggested that the lentiviral vector transduction of BMSCs was highly efficient and stable.

### 3.2. Regulation of Hippo Signaling by High and Low Expression of* Lats1* and* Lats2*

**Figure 3:**
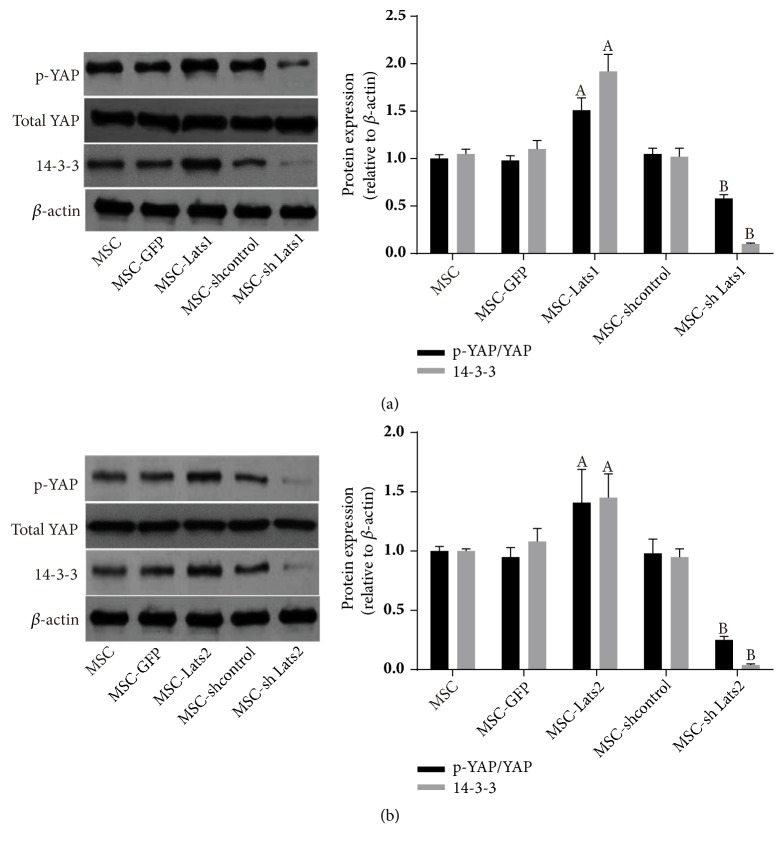
**Regulation of Hippo signaling in BMSCs after* Lats1* and* Lats2* genetic modification mediated by lentiviral vector transduction**. The expressions of YAP and 14-3-3 protein in BMSCs after transduction of* Lats1* (a) and* Lats2 *(b) were evaluated by Western blot (n = 3;  _ _^A^* P* < 0.05 vs. MSC-GFP,  _ _^B^* P* < 0.05 vs. MSC-shcontrol).

The phosphorylated yes-associated protein (p-YAP), a downstream cotranscriptional activator of* Lats1* and* Lats2*, and the protein level of 14-3-3 were determined by western blot to evaluate the regulatory effects of* Lats1* and* Lats2* on Hippo signaling. The results suggested that the levels of p-YAP and 14-3-3 protein of the MSC-*Lats1* and MSC-*Lats2* group were significantly higher than those of the MSC-GFP group, whereas the levels of p-YAP and 14-3-3 protein of the MSC-sh*Lats1* and MSC-sh*Lats2* group were significantly lower than those of the MSC-shcontrol group. However, differences in p-YAP and 14-3-3 protein levels between the MSC, MSC-GFP, and MSC-shcontrol groups were not significant (Figures 3(a) and 3(b). These results suggested that elevated and decreased expression of* Lats1* and* Lats2* could activate and inhibit the Hippo signaling pathway, respectively.

### 3.3. Effects of High and Low Expression of* Lats1* and* Lats2* on the Osteogenic and Adipogenic Differentiation of BMSCs

**Figure 4:**
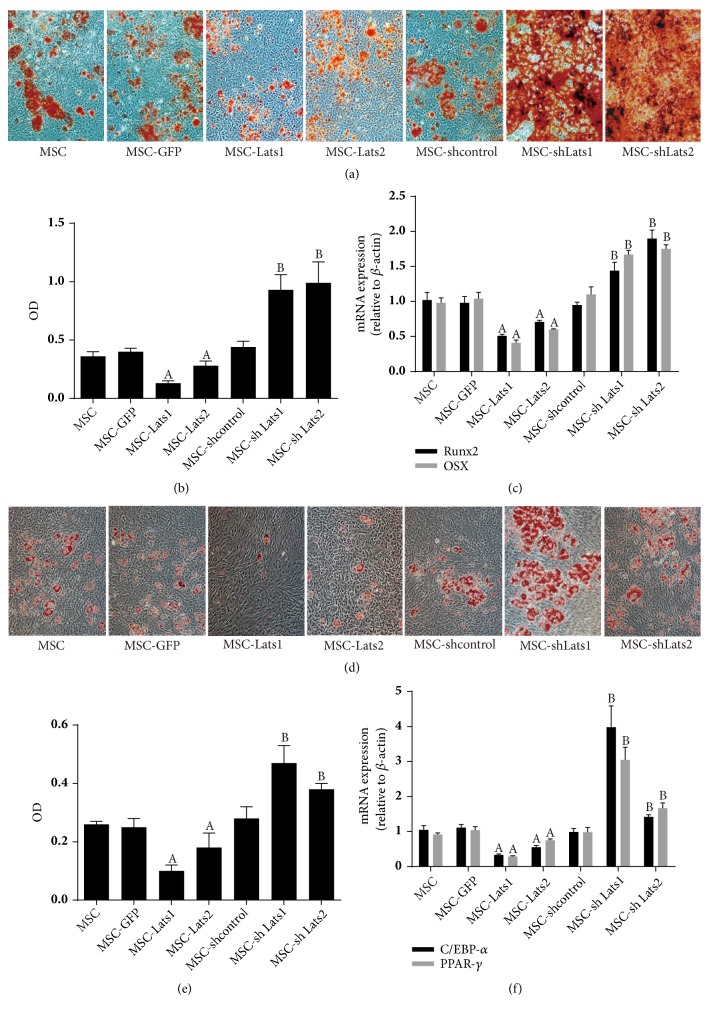
**The effects of high and low expression of* Lats1* and* Lats2 *on the osteogenic and adipogenic differentiation of BMSCs**. Mineralized bone matrix was visualized with Alizarin Red S staining ((a), 200×), Alizarin Red S was extracted from cells with cetylpyridinium chloride, and optical density (OD) at a wavelength of 570 nm was then measured (b), and osteogenic-specific gene (Runx2, OSX) expression was evaluated by qRT-PCR (c). The accumulation of neutral lipid vacuoles was visualized by Oil red O staining ((d), 200×), Oil red O was extracted from cells with isopropanol and optical density (OD) at a wavelength of 520 nm was then measured (e), and adipogenic-specific gene (C/EBP*α*, PPAR-*γ*) expression was evaluated by qRT-PCR (f) (n = 3;  _ _^A^* P* < 0.05 vs. MSC-GFP,  _ _^B^* P* < 0.05 vs. MSC-shcontrol).

The effects of* Lats1* and* Lats2* on the osteogenic differentiation of BMSCs were determined using Alizarin Red S quantification of calcium deposits, along with the detection of mRNA expression of the osteogenic cell-specific transcription factors, Runx2 and OSX, by RT-PCR. The results showed that calcium deposition and* Runx2* and* OSX* expression of the MSC-*Lats1* or MSC-*Lats2* group were significantly lower than those of the MSC-GFP group, whereas these measures in the MSC-sh*Lats1* or MSC-sh*Lats2* group were significantly higher than those of the MSC-shcontrol group. Differences in these measures between the MSC, MSC-GFP, and MSC-shcontrol groups were not significant (Figures 4(a), 4(b), and 4(c)). Hence, the above results suggested that increased and decreased expression of* Lats1* and* Lats2* could inhibit and promote the osteogenic differentiation of BMSCs, respectively.

Oil red O staining was performed to quantify accumulation of neutral lipid vacuoles and qRT-PCR analysis was used to detect the mRNA expression of the adipogenic cell-specific transcription factors, C/EBP*α* and PPAR-*γ*, to assess the effects of* Lats1* and* Lats2* on adipogenic differentiation of BMSCs. The results suggested that accumulation of lipid vacuoles and the mRNA expression of* C/EBPa* and* PPAR-γ *of the MSC-*Lats1* or MSC-*Lats2* group were significantly lower than those of the MSC-GFP group, whereas these measures of the MSC-sh*Lats1* or MSC-sh*Lats2* group were significantly higher than those of the MSC-shcontrol group. The levels of these measures did not differ between the MSC, MSC-GFP, and MSC-shcontrol groups (Figures 4(d), 4(e), and 4(f)). The above results indicate that increased and decreased expression of* Lats1* and* Lats2* could inhibit and promote the adipogenic differentiation of BMSCs, respectively.

### 3.4. Effects of High and Low Expression of* Lats1* and* Lats2* on the Proliferation of BMSCs

**Figure 5:**
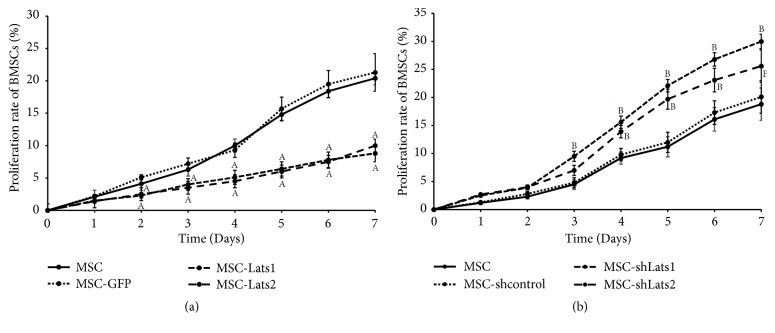
**The effects of high and low expression of* Lats1* and* Lats2* on the proliferation of BMSCs**. The growth curves of the BMSCs were evaluated by the CCK-8 assay after transduction for 7 days. The proliferation rate of the MSC-*Lats1* and MSC-*Lats2* group was significantly lower than the MSC-GFP group from days 2 to 7 (a). The proliferation rates of the MSC-sh*Lats1* and MSC-sh*Lats2* group were significantly higher than the MSC-shcontrol group from days 3 to 7 and from days 4 to 7, respectively (b). The results are representatives of three independent experiments (n = 3;  _ _^A^* P* < 0.05 vs. MSC-GFP,  _ _^B^* P* < 0.05 vs. MSC-shcontrol).

The effect of* Lats1* and* Lats2* on BMSCs proliferation was measured using the CCK-8 assay. The results indicated that the cell proliferation of the MSC-*Lats1* and MSC-*Lats2* group were significantly lower than that of the MSC-GFP group, with the effect being most obvious from days 2 to 7 (Figure 5(a)). Conversely, the cell proliferation of the MSC-sh*Lats1* and MSC-sh*Lats2* group was significantly higher than that of the MSC-shcontrol group, with the effect being most obvious from days 3 to 7 and from days 4 to 7, respectively (Figure 5(b)). In addition, the degrees of cell proliferation did not differ among the MSC, MSC- GFP, and MSC-shcontrol groups (Figures 5(a) and 5(b)). These results indicate that increased and decreased expression of* Lats1* and* Lats2* could inhibit and promote BMSCs proliferation, respectively.

### 3.5. Effects of High and Low Expression of* Lats1* and* Lats2* on the Migration of BMSCs

**Figure 6:**
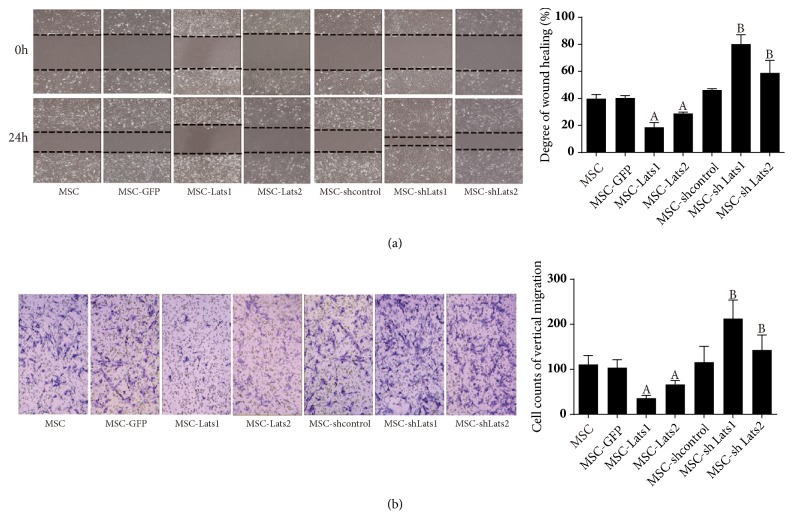
**The effects of high and low expression of* Lats1* and* Lats2* on the migration of BMSCs**. The horizontal migration of BMSCs was determined by the scratch assay (a). The wound width was observed and imaged at 0 and 24 h (200×). The degree of wound healing was shown as bar graph. The vertical migration of BMSCs was determined by the Transwell migration assay (b). The migrating cells below the insert membrane were stained with crystal violet and observed by an inverted phase-contrast microscope (200×). The cell count was shown as bar graph (n = 3;  _ _^A^* P* < 0.05 vs. MSC-GFP,  _ _^B^* P* < 0.05 vs. MSC-shcontrol).

The effects of* Lats1* and* Lats2* on the horizontal migration of BMSCs were assessed using a scratch assay. The results indicated that the wound width of the MSC-*Lats1* or MSC-*Lats2* group was significantly larger than that of the MSC-GFP group, whereas the wound width of the MSC-sh*Lats1* or MSC-sh*Lats2* group was significantly smaller than that of the MSC- shcontrol. The differences in wound width among the MSC, MSC-GFP, and MSC-shcontrol groups were not significant (Figure 6(a)). These results indicated that increased and decreased expression of* Lats1* and* Lats2* could inhibit and promote the horizontal migration of BMSCs, respectively.

A Transwell migration assay was performed to assess the effects of* Lats1* and* Lats2* on the vertical migration of BMSCs. The results showed that the number of cells that migrated to the compartment below the Transwell membrane of the MSC-*Lats1* or MSC-*Lats2* group was significantly lower than that of the MSC-GFP group. Conversely, the number of migrating cells of the MSC-sh*Lats1* or MSC-sh*Lats2* group was significantly higher than that of the MSC-shcontrol group. In addition, there was no significant difference in the number of migrating cells between the MSC, MSC-GFP, and MSC-shcontrol groups (Figure 6(b)). These results indicated that increased and decreased expression of* Lats1* and* Lats2* inhibited and promoted the vertical migration of BMSCs, respectively.

## 4. Discussion

The main pathophysiological changes in ARDS comprise alveolar epithelial cell damage followed by the proliferation and differentiation of the remaining alveolar epithelial type II (ATII) cells into alveolar epithelial type I (ATI) cells to repair the lung injury [9]. However, in moderate and severe ARDS, ATII cells are severely damaged, limiting the self-repair effect; therefore, an exogenous supply of seed cells becomes a better therapeutic choice. Notably, BMSCs may serve as ideal seed cells in the repair of alveolar epithelial cells in ARDS. Studies have demonstrated that exogenous BMSCs helped to accelerate the repair of alveolar epithelium in animal models of ARDS, attenuating the lung injury in ARDS and reducing mortality [10, 11]. However, in ARDS, the number of BMSCs homing to the lung is low, their retention time in the lung is short, and the percentage of BMSCs differentiating into lung ATII cells is also low, therefore limiting the overall effect of these cells in alleviating ARDS [5, 6]. Thus, clarification of the regulatory mechanism of MSC differentiation, proliferation, and migration may assist in improving the lung protection effect of BMSCs [12].

In particular, the Hippo signaling pathway comprises an important means of regulating cell differentiation and proliferation, of which several ligands, receptors, kinases, transcriptional regulators, and transcription factors are expressed in BMSCs. The Hippo signaling pathway may therefore theoretically influence the biological behaviours of BMSCs including differentiation, proliferation, and migration [13]. In mammals, Hippo signaling is initiated by the binding of the cell surface ligand Dach-sous (Dchs) 1/2 to the adjacent cell surface receptor FAT tumour suppressor homolog (Fat) 4, which activates the STE20 family kinases, mammalian sterile 20-like kinases (MST) 1 and 2. With the aids of linker protein Salvador (SAV) 1 and Mps one binder kinase activator-like 1 (MOBKL1) A and B, MST1/2 kinases phosphorylate and activate the NDR family kinases* Lats1* and* Lats2* [14]. The primary target of* Lats1* and* Lats2* kinases is the transcriptional regulator YAP.* Lats1* and* Lats2* kinases phosphorylate YAP, which then bind to 14-3-3 protein resulting in its relocation to the cytoplasm and the inhibition of the proproliferation and antiapoptotic activities of YAP, thereby promoting cell death. If* Lats1* and* Lats1* activity decrease, YAP is not phosphorylated and thus enters into the nucleus. The YAP accumulated in the nucleus can bind to transcription factors such as TEAD1, which in turn promotes the expression of downstream target genes, thereby initiating the proproliferation and antiapoptotic activities of YAP/TAZ, promoting cell proliferation [15]. Hence, regulation of* Lats1* and* Lats2* constitutes a feasible approach to modulate the Hippo signaling pathway.

The present study utilised recombinant lentiviral vectors to transduce BMSCs with* Lats1*,* Lats2*, and the corresponding shRNA sequences and successfully constructed BMSCs cell lines that stably expressed* Lats1* and* Lats2* at high or low levels. Through the high expression of* Lats1* and* Lats2*, the key signaling molecules in the Hippo signaling pathway and their downstream transcription regulators, YAP and TAZ, were phosphorylated and activated, which in turn activated Hippo signaling, inhibiting its proproliferation and antiapoptotic activities [16]. Low expression (inhibition) of* Lats1* and* Lats2* could reduce YAP and TAZ phosphorylation, promoting YAP/TAZ localization to the nucleus and binding to TEAD, thereby inhibiting Hippo signaling, activating its proproliferation and antiapoptotic activities [17]. In the present study, the high and low expression of* Lats1* and* Lats2* successfully activated and inhibited the Hippo signaling pathway in BMSCs, respectively.

We further found that activation of Hippo signaling could inhibit the osteogenic and adipogenic differentiation of BMSCs, whereas inhibition of Hippo signaling promoted such cell behaviour. These results differed from previous findings. For example, a study by An et al. indicated that Hippo signaling is a positive regulator in adipose development. Under adipogenic culture conditions, the activation of Hippo signaling in BMSCs increased adipocyte differentiation [18]. However, Chen et al. found that, under osteogenic culture conditions, the activation of Hippo signaling in BMSCs inhibited its osteogenic differentiation [19]. In addition, Tang et al. reported that, under myogenic culture conditions, Hippo signaling activation could promote the myogenic differentiation of BMSCs [20]. Thus, the regulatory effects of Hippo signaling on BMSC differentiation may vary depending on the cell types, culture conditions, and time points [21].

Additionally, activation and inhibition of Hippo signaling were also shown to promote and inhibit BMSCs proliferation, respectively. These results also differed from previous findings. In particular, Ye et al. found that the activation of Hippo signaling promoted mouse ovarian stem cell proliferation [22]. In addition, a study by Lange et al. indicated that the blockage of Hippo signaling by knocking out Mst1/2 inhibited the proliferation of mouse airway epithelium progenitor cells [23]. However, An et al. found that, in mammals,* Lats1* overexpression activated Hippo signaling, which inhibited adipogenic cell proliferation [18]. Thus, the regulatory effects of the Hippo signaling pathway on cell proliferation may also vary greatly depending on cell types, culture conditions, and cellular microenvironment. Therefore, the regulatory effect of the Hippo signaling pathway on BMSC proliferation remains to be further elucidated.

Finally, the present study found that inhibition of the Hippo signaling pathway could enhance the vertical and horizontal migration of BMSCs, whereas activation of the Hippo signaling pathway reversed the above effects. Exogenous BMSCs can accumulate in the injured sites, which is a prerequisite for their effects of tissue repair. Previous studies have shown that, in animal model of ARDS, the number of BMSCs homing to ARDS lung tissues was significantly higher than that homing to the normal lung tissue. For example, Ortiz et al. found that, in a bleomycin-induced lung injury model, the amount of BMSCs migrating to the lung tissue was 23 times higher than that found in normal mice [24]. Currently, the molecular mechanism regulating MSC migration to the injured site remains controversial. It is believed that stem cell derived factors-1*α* and the chemokine receptor CXCR4 may play important roles [25]. In addition, other studies have suggested that TGF-*β*1, IL-1*β*, and TNF-*α* may induce BMSCs to secrete matrix metalloproteases to promote homing [26]. The results of our study demonstrate that the Hippo signaling pathway could regulate MSC migration* in vitro*, providing new perspective for the regulatory mechanism of MSC migration and homing. However, the* in vivo* regulatory effects and mechanisms of the Hippo signaling pathway deserve further investigation.

In conclusion, our study found that reduced expression of* Lats1* and* Lats2* inhibited Hippo signaling and promoted the* in vitro *osteogenic and adipogenic differentiation, proliferation, and migration of BMSCs. Inhibition of Hippo signaling in BMSCs may help enhance their role in tissue repair in ARDS, although further* in vivo* studies are required.

## Data Availability

All data generated or analyzed during this study are included in this article.
